# Always in (partner) action? Working in teams may improve simulated flight performance—but only in the apt cognitive control state

**DOI:** 10.1186/s41235-025-00633-6

**Published:** 2025-06-02

**Authors:** Sophie-Marie Stasch, Wolfgang Mack, Yannik Hilla

**Affiliations:** Universität Der Bundeswehr München, Werner-Heisenberg-Weg 39, 85579 Neubiberg, Germany

**Keywords:** Multitasking, Cognitive control, Teamwork, Flight environment

## Abstract

Multitasking abilities are vital for conducting flight missions. Traditional theories of multitasking suggest that cognitive resources represent a determining factor of said performance. The current study takes a different approach by investigating how the stability-flexibility-dilemma of cognitive control influences multitasking performance in a simulated flight environment. Besides, we investigate how this dilemma interacts with performance and workload when an additional partner is present. For this purpose, 42 participants were recruited to perform the open-source version of the Multi-Attribute Task Battery (openMATB) in two different experimental conditions. Initially, participants performed the openMATB alone either in a stable or flexible control mode, which was manipulated via a gamification method (assessment 1). Afterward, two participants performed the openMATB together as a team – again in a stable and flexible control mode (assessment 2). Results indicate that the stability-flexibility-dilemma affected the participants’ individual task performance. Furthermore, the participants’ performance improved in teams. However, this effect depended on subtask characteristics and the operated cognitive control mode. Implications for the design of adaptive assistance systems and suggestions for future research are discussed.

## Multitasking in flight missions

Conducting (military) flight missions requires performing additional tasks in parallel to flying an aircraft: for instance, monitoring flight and weather parameters, responding to radio messages, and communicating with flight and ground control teams, while simultaneously looking out for potential threats. Such multitasking conditions impose high cognitive demands on pilots, causing performance decrements in the long run, which may potentially lead to hazardous incidents (Berman & Dismukes, 2006; Chérif et al., 2018; Kelly & Efthymiou, 2019; Loukopoulos et al., 2009). Unraveling why and how multitasking behaviour relates to such negative outcomes, although assistance in the form of a second pilot is often available, may help to prevent them.

### Theories on multitasking

The term *multitasking* was coined by the IT industry in the 1960s and refers to a computer system completing multiple tasks in parallel or in rapid succession (Burke, 2023). But the first experiments to investigate human multitasking abilities started much earlier in the 1930s, with Telford discovering the *psychological refractory period* (Telford, 1931). This phenomenon describes individuals’ impaired response capabilities to stimuli as a function of temporal overlap. The *central bottleneck theory* has been frequently used to explain this phenomenon (Smith, 1967). It suggests that information processing requires running through several stages, whereby only one stimulus may be processed at a time. Thus, processing multiple inputs simultaneously is not possible due to a capacity limit – or only possible in the case of interference at any processing stage so long as the capacity limit is not exceeded. However, which information processing stage predominantly relates to the psychological refractory period is still debated (Klapp et al., 2019).

Likewise, performance decrements related to multitasking are likely based on capacity limits. The most established theory to address this issue in the aviation domain is the *theory of multiple resources* by Wickens (1980). It proposes that performance declines as a function of resource expenditure beyond the capacity of different information processing modalities (auditory/visual) and codes (verbal/spatial), and has been successfully employed to predict how multitasking affects mental workload (Sarno, 1995) and performance (Wickens & Dixon, 2002). Working memory processing abilities likely represent determinants of said capacities (Oberauer & Kliegl, 2006). However, algorithmic solutions how to distribute resources during multitasking must also be considered in this context. This is especially important because resource-centric models of multitasking, such as Wickens’ (1980) multiple resource theory, primarily offer a descriptive framework for explaining performance decrements rather than a mechanistic account for those. While these models intuitively capture multitasking limitations, recent research suggests that such constraints may stem may reflect a balance between learning efficacy and processing efficiency with cognitive control playing a vital role in this process (Musslick & Cohen, 2021).

The *theory of event coding* (Hommel & Wiers, 2017), for instance, points out that performance depends on associations between perceptions and actions represented as so-called *event files*. Thus, multitasking decrements may emerge as a result of event files competing for resources, whereby events files are thought to have mutual inhibitory connections. The competition between event files is resolved by metacontrol (Hommel, 2020). Metacontrol minimizes or maximizes the mutual inhibition between event files, for instance, based on the top-down influence of the current goal (Hommel, 2015). In the case of high mutual inhibition of event files, metacontrol is related to cognitive persistence while a low mutual inhibition relates to cognitive flexibility.

This balance between cognitive persistence, sometimes also called cognitive stability, and cognitive flexibility is called *stability-flexibility-dilemma of cognitive control* (Eppinger et al., 2021). On the one hand, cognitive flexibility is necessary to quickly switch between tasks and to adapt information processing to changing task demands. On the other hand, cognitive stability protects task execution from interference in the face of distractors. On the flip side, distractions from irrelevant stimuli can easily occur in a state of high cognitive flexibility. Likewise, cognitive stability is linked to increased task-switching costs (see Qiao et al., 2023; Fröber et al., 2018, on the role of the stability-flexibility-dilemma in multitasking; Musslick et al., 2019; Liljenström, 2003; Herd et al., 2014, for a computational framework thereof; and Dreisbach et al., 2005, for insights on the neurobiology related to the flexibility-stability dilemma).

Importantly, individuals’ abilities of resolving the stability-flexibility-dilemma appear to be related to the updating threshold of their working memory (Goschke & Bolte, 2014). Thus, a high threshold determines the stability of working memory representations and how easily novel information can access working memory. In other words, cognitive stability is linked to a high updating threshold, which, in turn, is associated with increased working memory resistance towards distractions and strengthens the pursuit of goals. Likewise, new information cannot enter working memory as easily as compared to a low updating threshold. In contrast, cognitive flexibility is linked to a low updating threshold, which facilitates task-switching but also increases the likelihood of task interference and distraction because new information can easily access working memory.

### Shared mental models in safety–critical multitasking environments

How individuals deal with the stability-flexibility-dilemma may predict their performance in multitasking environments. In support of this, previous research showed that manipulating individuals’ cognitive control modes (stable vs. flexible) using a gamification approach (Stasch & Mack, 2023b) affected their performance in simulated flight environments (Stasch & Mack, 2023a).

However, these results were found in individuals performing on their own, while flight missions are often carried out by at least two individuals. Multi-role fighter jets, like the *Boeing F/A-18E/F Super Hornet* or the *Eurofighter Typhoon T3*, can have a two-set configuration, in which two pilots sit behind each other. In these configurations, the front-seat pilot is commonly occupied with tasks like flight control, navigation, and communication with air traffic control, while the rear-seat pilot may operate and manage onboard weapons and electronic warfare systems or monitor sensors in a reconnaissance mission. Similar conditions can be observed in commercial aircrafts: the *Pilot Flying* is primarily responsible for piloting the aircraft while the *Pilot Monitoring* monitors the flight management and is responsible for all other tasks, like communication with Air Traffic Control. Thus, both pilots have distinct roles with clearly defined tasks following standard operating procedures. Hereby, shared mental models are a key factor in the successful coordination of flight tasks representing *“knowledge structures held by members of a team that enable them to form accurate explanations and expectations for the task, and, in turn, coordinate their actions and adapt their behavior to demands of the task and other team members”* (Cannon-Bowers et al., 1993, p. 228). Mathieu et al. (2000), for instance, demonstrated that the similarity of knowledge structures between two team members successfully predicted the quality of team processes and performance in a flight combat simulator. Besides, Tremblay et al. (2012) showed that collaboration in a functional dyad improved individuals’ performance in a command-and-control environment compared to individuals working alone. Thus, working in teams appears to improve performance in complex multitasking environments.

A determining factor for this effect may be the distribution of workload. Perceived workload during a solitary performance may fundamentally differ from the one experienced during teamwork, given that teamwork requires cooperative efforts, for instance, communication, coordination, monitoring, and dealing with (leadership) hierarchies (Bowers & Salas, 1997). Thus, team members may both support and hinder an effort depending on the overall workload generated by a task in relation to the resources contributed by each member of a team. Serfaty et al. (1993) found that communication was reduced in high-workload environments, which Urban et al. (1996) interpreted as a coping strategy to offset the consequences of the high-workload scenario. Consequently, co-workers might place additional strain on the mental workload and represent a cost.

### Hypotheses

This raises the question if and how the stability-flexibility dilemma affects multitasking performance in teams? We addressed this question by recruiting 42 officer cadets to perform simulated flight tasks either in a stable or flexible cognitive control mode, and either on their own (solitary) or with a partner (as a team). Based on previous research by Stasch and Mack (2023a), it was hypothesized that the cadets will demonstrate a better task performance in a primary task (flying an aircraft) and worse performance in secondary tasks (monitoring, listening to radio messages, and fuel resource management), while employing a *stable* cognitive control state (*H1a*). In contrast, secondary task performance should increase as a result of using a flexible cognitive control state at the expense of worse task performance in the primary task (*H1b*). Besides, based on findings reported by Mathieu et al. (2000) and Tremblay et al. (2012), it was expected that the cadets would benefit from a partner’s aid such that their steering performance may improve even further in a stable cognitive control condition (*H2a*), and performance decrements in secondary tasks accompanied with a stable cognitive control mode may be compensated (*H2b*). Likewise, primary and secondary task performance should be enhanced in teams compared to single task performance in case a flexible cognitive control mode was employed (*H2c, H2d*). Moreover, as elaborated above, alterations in mental workload constitute a vital factor in successful teamwork performance (Bowers & Salas, 1997). Thus, we also expected correlations between perceived mental workload and performance. Furthermore, we anticipated that personality and social traits may impact on multitasking performance in teams: Himi et al. (2023) for instance found that the personality traits conscientiousness, openness to experiences, and polychronicity (i.e., the preference for performing several tasks at once) may moderate the relationship between cognitive abilities and multitasking behavior. Additionally, neuroticism and conscientiousness appear to affect team performance (Vîrgă et al., 2014). Aside this, communication, collaboration, and perspective taking/self-reflection skills and trust in a partner’s competence may play a vital role in successful teamwork (Mills et al., 2015; Perterer et al., 2019). Therefore, we also controlled for correlations between these constructs and performance.

## Methods

### Sample

We conducted power simulations to estimate the required sample size for finding reliable conditional performance differences (see *Sample Size Analysis*, for a more detailed description). This procedure suggested that data of at least 29 individuals were required. We collected data of 42 participants. We gathered more data than indicated by our sample size analysis to compensate for potential dropouts, which were likely due to participants having to attend two experimental test sessions, and to obtain more reliable estimates. Indeed, two of them did not participate in all assessment conditions, and two additional participants did not display sufficient performance. Thus, 38 datasets were analyzed. Except for two civilian individuals, all participants were officer cadets enrolled as students at the University of the Bundeswehr Munich (Germany). Thus, our sample differed from typical university students, as participants had completed approximately 15 months of basic military training and were selected through an assessment center for the officer program. They were between 20 and 30 years of age (*M* = 22.81, *SD* = 2.82, *M*_Bayes_ = 23.04, 95% CI [22.03, 24.03], Age ~ exponentially modified Gaussian Distribution). The majority represented undergraduate students (*n* = 34), who pursued studies in Social Sciences (*n*_Psychology_ = 31), identified as male (*n* = 25) and were right-handed (*n* = 33). Moreover, 14 participants played video games (*M* = 9.68, *SD* = 9.66, range [1, 36], *M*_Bayes_ = 6.76, CI [4.64, 8.88], Play Time ~ exponentially modified Gaussian Distribution). Regarding the participants’ military background, the majority were affiliated to the Infantry (*n* = 20), had already served for at least one year (*M* = 3.01, *SD* = 1.26, range [1, 8], *M*_Bayes_ = 3.79, CI [3.2, 4.36], Military Service Duration ~ exponentially modified Gaussian Distribution), and 14 of them had flight experience (in hours) (*M* = 15.43, *SD* = 10.84, range [4, 59], *M*_Bayes_ = 11.7, CI = [9.41, 13.91], Flight Experience in hours ~ exponentially modified Gaussian Distribution). We did not offer a monetary compensation but participants could acquire student lab tokens for participation. All participants provided written informed consent prior to assessments. Moreover, this study was approved by the local Ethics Committee (EK UniBw M 23–16).

### Procedure and methods

#### Low-fidelity flight simulator (multi-attribute task battery)

The participants performed the open-source version of the *Multi-Attribute Task Battery* (openMATB; Cegarra et al., 2020), which represents a low-fidelity flight simulator. It requires individuals to execute four flight-like tasks simultaneously: in the *tracking* task, individuals need to steer a circle as close to the center of a hair cross as possible using a joystick, which resembles flying an aircraft. For this, a target radius of 0.175 units was used in the scenario script. In the *system monitoring* task, individuals need to monitor four barometers and two buttons. If arrow indicators deviate too strongly from the midlines of barometers either F1, F2, F3, or F4 must be pressed. Moreover, if buttons change color, F5 or F6 must be pressed, respectively. The scenario script contained six events per trial for the system monitoring task: four barometer failures and two button failures that required a F5 or F6 press. In the *communications* task, individuals need to listen and respond to radio messages. These comprise instructions as to which individuals are supposed to change the frequency of one of four radios, using arrow keys. Per trial, the scenario script contained two radio messages directed to the participants (own) and two radio messages directed to other aircrafts (other). In the *resource management* task, individuals must control the fuel levels of two tanks by either dis- or enabling pumps using number keys. Throughout one trial, the pump status changed nine times: in five instances, a pump unexpectedly turned on; in two instances, a pump turned off; and in two other instances, a pump failed. The optimal levels were set to 2000 units for tank A and 2500 units for tank B, with both tanks losing 1100 units per minute. Parameters of the scenario script were adopted from previous research conducted by Stasch and Mack (2023a, b). One trial lasted for 90 s. Additionally, two different scenarios (scenario A and scenario B) were created that varied the temporal procedure of events, but always contained the same number and type of events. Those scenarios were used to prevent practice effects.

Besides, the participants performed the openMATB in four different conditions, whereby the cognitive control mode to execute tasks (stable vs. flexible) and the performance mode (solitary performance vs. team) differed. Solitary and team performance were assessed separately.

#### Solitary performance

In the first assessment round, the participants performed the openMATB by themselves. At first, they practiced each openMATB task separately for one minute, and subsequently in conjunction for approximately seven minutes. Following this, their solitary openMATB performance assessments started. These comprised four blocks of five trials, whereby either a stable or flexible cognitive control mode was supposed to be employed during task execution, respectively. Additionally, participants performed each cognitive control mode condition in scenario A and scenario B. The cognitive control mode was elicited by means of a gamification method developed by Stasch and Mack (2023b). Hereby, the participants’ cognitive control mode is altered by information about weather conditions and corresponding task prioritization instructions, and feedback regarding their performance. In good weather conditions, individuals are instructed to pay attention to each openMATB task equally, which puts them in a *flexible* cognitive control mode. In contrast, in bad weather conditions, individuals are instructed to prioritize the tracking task over the remaining tasks, eliciting a *stable* cognitive control mode. After each trial, the participants receive a feedback score (varying between 0 and 100), which rewards either switching between tasks (flexible cognitive control mode) or attending to one primary task (stable cognitive control mode). The feedback score is calculated based on the number of task switches during a trial. A task switch was identified when a fixation moved from one Area of Interest (AOI) to another and were then normalized using baseline values derived from previous in-house experiments. The flexible control mode was reinforced when a high number of task switches occurred, whereas the stable control mode was reinforced when task switches were minimal. This approach aligns with Dreisbach and Fröber (2019), who associate increased task switching with a flexible control mode and reduced switching with a stable control mode.

The participants performed each cognitive control condition in two blocks (based on five trials of scenario A and scenario B), respectively. To control for order effects, half of participants performed the four blocks in experimental sequence 1 (ScenarioA_flexible_ – ScenarioB_stable_ – ScenarioA_stable_ – ScenarioB_flexible_), while the other half of participants performed the four blocks in experimental sequence 2 (scenarioB_stable_ – scenarioA_flexible_ – scenarioB_flexible_ – scenarioA_stable_). Thus, by the end of the first assessment, the participants acquired a strong representation of which cognitive control mode to employ in response to the respective weather conditions (Eppinger et al., 2021).

#### Team performance

**Fig. 1:**
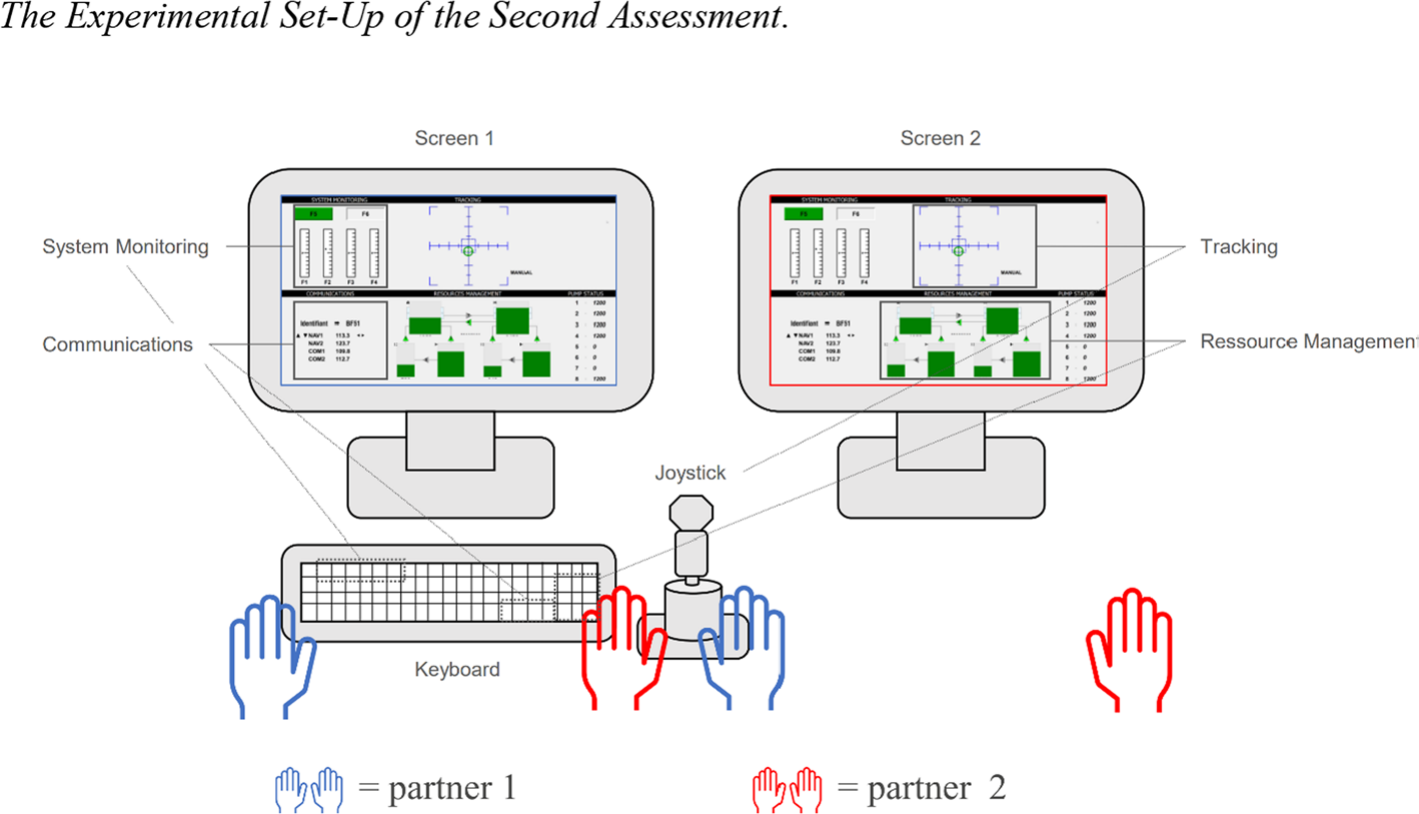
The experimental set-up of the second assessment*. Note.* The participants performed the open-source version of the Multi-Attribute Task Battery (openMATB; Cegarra et al., 2020) in four conditions: either employing a stable or a flexible cognitive control mode, and either by themselves or in teams of two. The openMATB comprises four subtasks (tracking, system monitoring, communications, and resource management) to simulate flight performance. In the stable control condition, participants were to focus on the tracking task (flying the aircraft). In the flexible condition, they were to focus on all tasks equally well. When performing in teams, participants only performed the tracking task manually together, but the front-seat partner (blue) executed all remaining tasks, while the rear-seat partner was only allowed to verbally support (red)

Subsequently, two participants randomly formed a team to perform the openMATB in the two different control modes again. However, this time, no feedback (gamification method) was presented. For performing the openMATB in teams, the participants sat side-by-side at a table with two screens mounted in front of them (see Fig. 1). Both screens displayed the same image (that is one screen mirrored the image of the other). The tracking task was performed by both participants concurrently and throughout the assessment time. The remaining tasks required *Partner 1* (left side) to manually respond. *Partner 2* (right side), in turn, was not allowed to manually respond but was allowed to verbally support *Partner 1*. For a visualization of the set-up, see Fig. 1. We designed the set-up like this for two major reasons: firstly, in doing so, we simulated work conditions as experienced by pilots in cockpits as *pilots flying* and *pilots monitoring* (see Introduction section). Additionally, steering the joystick together throughout the experiment ensured that the participants actually performed multitasking and teamwork continuously. Besides, a *pilot monitoring* can take over piloting if requested to do so. Thus, sharing the tracking task was a reasonable design choice for our research aim. Each participant performed the openMATB two times in each role and either in a block of good or bad weather conditions. Thus, every participant applied a different cognitive control model (flexible vs. stable) in a different scenario (A & B). For this, they switched places after two blocks had been performed – meaning that each individual performed two openMATB blocks of different scenarios as *Partner 1* and *Partner 2*, respectively. Thus, we acquired performance data of each individual as *Partner 1* two times. The teams were allowed to discuss strategies and to make breaks between blocks. Thus, they could discuss how they wanted to steer the joystick together and how they wanted to be verbally reported both before, after, and in-between task execution.

#### Task load

Besides, after each block, the participants reported how much workload they had perceived during task execution, using the *NASA task load questionnaire* (Hart & Staveland, 1988). In detail, they indicated how much *mental*, *temporal*, and *physical* workload, how much *effort* and *frustration* they had experienced, and how proficiently they evaluated their *performance* while executing the openMATB, using a 21-point Likert scale. These scores were then used to compute a *NASA task load index* (TLX) (see Eq. (1)).

1 $$ TLX_{i} = \sum\limits_{j = 1}^{J} {\frac{{w_{ij} \times {\text{load}}\;{\text{value}}_{ij} }}{{\sum\nolimits_{j = 1}^{J} {{\text{load}}\;{\text{value}}_{ij} } }}} $$

Hereby, *TLX*_*i*_ denotes the perceived task load of an individual *i* as a function of the weighted average over their responses to each criterion *j* (with *J* = {mental load, temporal load, physical load, effort, frustration, performance}). Performance scale scores were reversely re-coded. Task load weights, *w*, of each criterion, *j*, for each individual, *i*, were estimated as described in Eq. (2),

2 $$ w_{ij} = \frac{{{\text{load}}\;{\text{value}}_{ij} }}{{\overline{{{\text{load}}\;{\text{value}}_{j} }} }} $$

With $\overline{{{\text{load}}\;{\text{value}}_{j} }}$ representing the expected value of the poisson distributed load values of criterion *j* (based on all individuals). *TLX*_*i*_ ~ 1 indicates that participants perceived task load equally demanding as the average over all participants, *TLX*_*i*_ > 1 that they perceived more task load in at least one criterion *j*, and *TLX*_*i*_ < 1 the opposite.

We used these formulars to compute the perceived task load for each individual, *i*, when performing the openMATB in the stable and flexible condition and by themselves (*solitary*) and as *Partner 1* in teams (*team)*. In the latter condition, their responses could have been affected by their partner as they were allowed to discuss the perceived workload. But given that the participants were allowed to discuss strategies throughout task performance and their perception of task load can be factored into these discussions anyways, we do not consider this a remarkable issue.

### Follow-up questionnaires

After performing the openMATB, the participants were asked to fill in questionnaires assessing their *demographics* (sex, handedness, education, age in years, video gaming habits, study subject (if applicable)), *military-specific characteristics* (if applicable) (military unit, military service in years, flight experience in hours and based on licenses), *personality traits* (extraversion, agreeableness, conscientiousness, neuroticism, openness to experiences, polychronicity), *social traits* (self-control, affinity for collaboration, communication style), and how proficient they rated their *partner’s competency*. We selected these personality and social traits for their role in multitasking (Himi et al., 2023) and team performance (Mills et al., 2015; Perterer et al., 2019; Vîrgă et al., 2014), respectively. In detail, for assessing the personality traits *extraversion*, *agreeableness*, *conscientiousness*, *neuroticism*, and *openness to experiences*, the German short-version of the *Big Five Inventory* was used (BFI-K; Rammstedt & John, 2005); and for assessing the participants’ personality trait *polychronicity* we used the *Inventory of Polychronic Values* (Bluedorn et al., 1999). As for social traits, we assessed the participants’ self-control/monitoring in social contexts using the German adaptation of the *Self-Monitoring Scale* (Collani & Stürmer, 2009; Snyder, 1974), which comprises four subscales (*acting*, *other directedness*, *sensitivity*, and *extraversion*) indicative of how susceptible individuals respond to or aim to affect social interactions. Moreover, we assessed their affinity for collaboration using the German adaptation of the *Collective Orientation Scale* (Driskell et al., 2010; Hagemann, 2020); and their communication style as a function of *impression manipulativeness*, *emotionality*, and *expressiveness* based on an English translation of the Italian short version of the *Communication Style Inventory* (Diotaiuti et al., 2020; Vries et al., 2013). Aside this, the participants could indicate how competent in terms of *expertise*, *integrity*, and *benevolence* they rated their partner, using the *Muenster Epistemic Trustworthiness Inventory* (Hendriks et al., 2015, 2017). We controlled for the fit between questionnaire model and data using structural equation modeling. To resolve convergence issues, some questionnaire items had to be excluded. The model fits thereof were acceptable given the sample size. Detailed information on the procedure, the outcomes of these analyses and descriptive statistics on the questionnaires can be found in the Supplementary Material on Open Science Framework (https://osf.io/mbp43/).

### Statistical methods

All statistical analyses were conducted using *RStudio* (R Core Team, 2024) and the following packages: *brms* (Version 2.20.4; Bürkner, 2017), *dplyr* (Version 1.1.3; Wickham et al., 2023a), *ggplot2* (Version 3.4.4; Wickham, 2016), *lavaan* (Version 0.6–16; Rosseel, 2012), *lme4* (Version 1.1–35.1; Bates et al., 2015), *mgcv* (Version 1.9–1; Wood, 2004), *patchwork* (Version 1.1.3; Pedersen, 2023), *RColorBrewer* (Version 1.1.3; Neuwirth, 2022), *simsem* (Version 0.5–16; Pornprasertmanit et al., 2021), *tidygam* (Version 0.2.0; Coretta, 2023), and *tidyr* (Version 1.3.0; Wickham et al., 2023b).

#### Sample size analysis

**Fig. 2:**
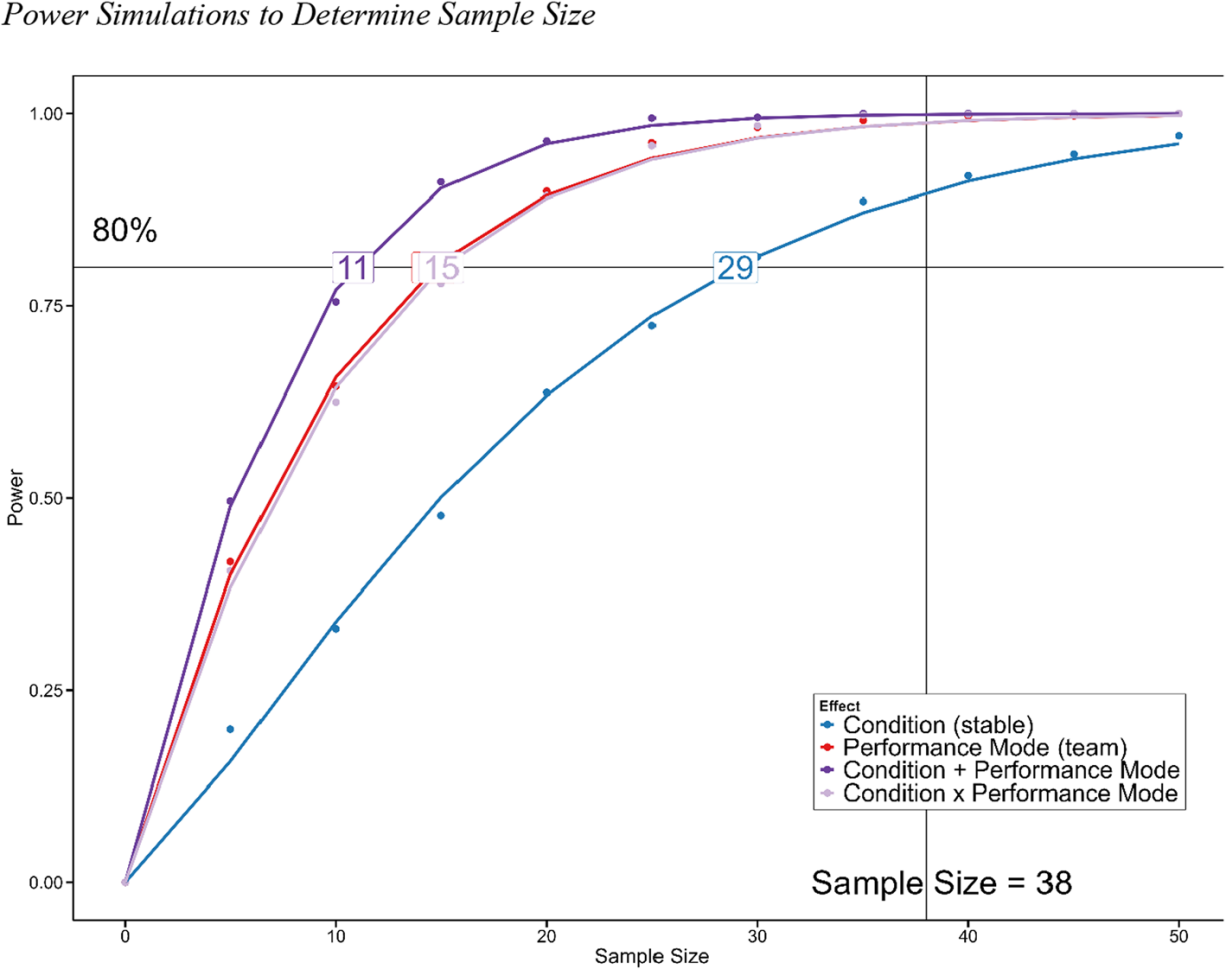
Power simulations to determine sample size. *Note.* Power estimations were made based on the number of hit outcomes (that is, if a hierarchical regression analysis indicated a main or interaction effect compared to an intercept-only model as a function of a Bayes factor > 3) relative to the total number of 1000 simulations. Sample sizes (*N*) ranged from 5 to 50 data points (following a skewed Normal distribution). Sample sizes for any desired power (here 80%) were determined by solving cumulative Weibull functions. The required sample size for the smallest effect (Condition; indicated in blue hue) was 29. Statistical analyses were made based on data of 38 participants

To determine the sample size, the following procedure was employed: firstly, random data samples of tracking task performance (as operationalized by RMSE) for each condition, following a skewed normal distribution (tracking_single,flexible_ ∼ Skewed Normal(0.08, 0.01, 3), tracking_single, stable_ ∼ Skewed Normal(0.074, 0.01, 3), tracking_team, flexible_ ∼ Skewed Normal(0.072, 0.01, 3), tracking_team, stable_ ∼ Skewed Normal(0.064, 0.01, 3)) were generated. Model parameter values were deduced from previously collected in-house data, and mean differences between distributions referenced to Cohen’s *d* effect sizes. We expected a medium-sized condition effect, a large performance mode effect, and a small interaction effect. Sample sizes ranged from 5 to 50 data points. Secondly, hierarchical linear models with tracking performance as criterion variable, condition (flexible vs. stable) and performance mode (solitary vs. team) as predictors and ID as a random effect were fitted 1000 times based on each sample size, respectively. Thirdly, main and/or interaction effects were determined based on Bayes factors (*BF*) (> 3) comparing the Bayesian information criterion values of models with predictors to those of intercept-only models with only random effects (Wagenmakers, 2007). The statistical power was then determined based on the number of hit outcomes (models with *BF* > 3) relative to all simulations. Fourthly, the required sample size (here at 80%) was subsequently approximated by fitting said power estimations to a cumulative Weibull distribution, using a Nelder–Mead algorithm. Thus, parameter values of said Weibull function could be estimated and deterministically solved according to a power of 80%. For a visualization of the simulated power values and cumulative Weibull curves, see Fig. 2. We chose to base our sample size analysis on tracking task performance based on two major reasons: firstly, it was the only task affected by the stable performance instruction and thus vital for our investigation. Furthermore, the tracking task is the only task requiring a continuous response. Therefore, it is less prone to confounds by application of different strategies, which makes predicting effect sizes more reliable.

#### Hypothesis testing

To test our hypotheses, we computed Bayesian hierarchical multi-level regression analyses with openMATB subtask performance values as criterion variables, and condition (stable vs. flexible) and performance mode (solitary vs. team) as predictor variables. Besides, we considered random effects whereby intra-individual variations on the subject-level nested in corresponding teams were considered in the models. This allows to control for random variance related to e.g., practice/learning and variance between teams. Tracking performance was operationalized based on the RMSE (deviation from center point), resource management task performance based on the average absolute deviation from optimal filling levels, and system monitoring and communications task performance based on the average number of misses per block. Bayesian posterior distributions were sampled based on four Markov-Chains with at least 5000 iterations. Priors were chosen based on previously acquired in-house data and adjusted to achieve a proper sensitivity (that means a match between simulated and observed data). Link functions were either Gaussian, exponentially modified Gaussian, or skewed normal distributions (see Hilla et al., 2023, for more details). The prior parameters of the reference models (solitary performance in flexible control mode condition) for each task were as followed: tracking task performance ~ exponentially modified Gaussian distribution with μ ~ Normal(0.08, 0.01), σ ~ Normal(0.01, 0.025), λ ~ Normal(0.01, 0.025), σ_random_ ~ Normal(0, 0.025), β_Condition_ ~ Normal(− 0.006, 0.025), β_Performance Mode_ ~ Normal(− 0.008, 0.025), β_Interaction_ ~ Normal(− 0.002, 0.025); system monitoring task performance ~ exponentially modified Gaussian distribution with μ ~ Normal(2.75, 0.05), σ ~ Normal(0.05, 1), λ ~ Normal(2.5, 0.1), σ_random_ ~ Normal(0, 1), β_Condition_ ~ Normal(0.03, 1), β_Performance Mode_ ~ Normal(− 0.04, 1), β_Interaction_ ~ Normal(− 0.02, 1); resource management task performance ~ Gaussian distribution with μ ~ Normal(500, 200), σ ~ Normal(200, 50), σ_random_ ~ Normal(10, 10), β_Condition_ ~ Normal(120, 50), β_Performance Mode_ ~ Normal(− 160, 50), β_Interaction_ ~ Normal(− 80, 50); and communications task performance ~ exponentially modified Gaussian distribution with μ ~ Normal(2.5, 0.5), σ ~ Normal(0.5, 1), λ ~ Normal(0.5, 0.05), σ_random_ ~ Normal(0, 1), β_Condition_ ~ Normal(0.02, 1), β_Performance Mode_ ~ Normal(− 0.4, 1), β_Interaction_ ~ Normal(− 0.1, 1). Conditional differences were modeled with reference to Cohen’s *d* effect sizes. We expected a medium-sized condition effect, a large performance mode effect, and a small interaction effect. Effects were determined by comparing marginal log-likelihood values of predictor models to intercept only models (with random effects), and comparing marginal log-likelihoods of predictor models between each other to determine best models. *BF* values needed to be at least 3 times larger for a model to qualify as better as its reference model.

#### Exploratory and control analyses

Aside from explicit task performance measures, we also investigate if and how condition (stable vs. flexible) and performance mode (solitary vs. team) affected the participants’ perceived task load. Thus, we performed the same procedure as described above but with TLX scores as the dependent variable. In this regard, we chose the following priors: TLX ~ Gaussian distribution with μ ~ Normal(1, 0.25), σ ~ Normal(0.25, 0.075), σ_random_ ~ Normal(0, 0.025), β_Condition_ ~ Normal(0, 0.075), β_Performance Mode_ ~ Normal(− 0.2, 0.075), β_Interaction_ ~ Normal(0, 0.075). Besides, we tested if including TLX scores as a metric predictor contributed to explaining the participants’ task performance. For this, we chose β_TLX_ ~ Normal(0, 0.025), β_TLX_ ~ Normal(0, 1), β_TLX_ ~ Normal(0, 50), and β_TLX_ ~ Normal(0, 1) for tracking, system monitoring, resource management, and communications task performance as priors, respectively.

At last, we investigate if and how the participants’ interaction effect (that is the additional difference in performance related to the stable-team performance condition in comparison to the condition and performance mode effect differences) was related to any metric demographic (video gaming play time, age), military-related (military service duration) or questionnaire-related variables. For this, we fitted generalized additive regression models (allowing for fitting non-linear relations) with z-standardized interaction effect performance values and z-standardized predictor variables. Effects were determined based on *BF* values (> 3) as described by Wagenmakers (2007).

## Results

### Multitasking performance

#### Tracking task

**Fig. 3:**
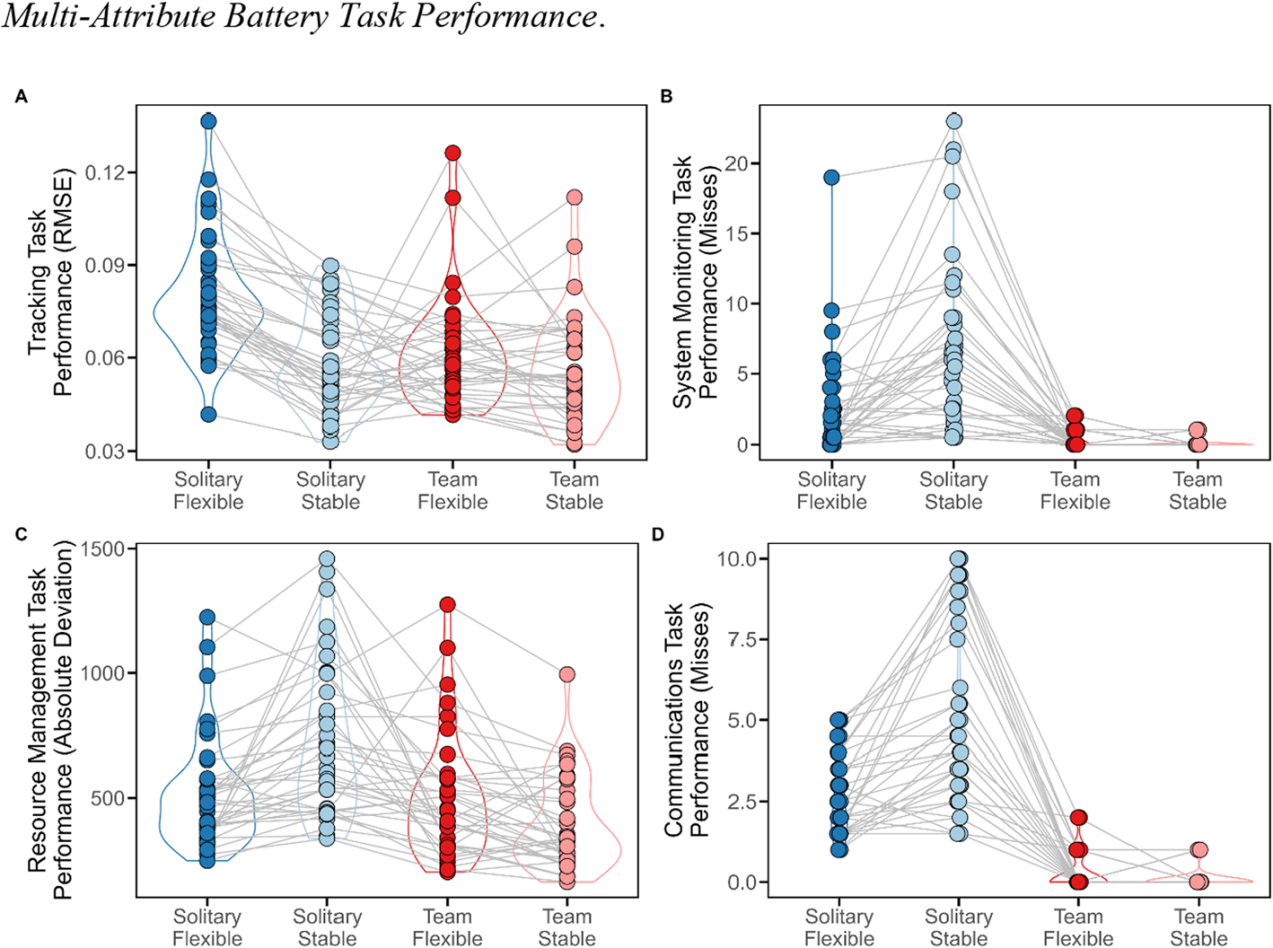
Multi-attribute battery task performance. *Note.* **A**. Tracking Task: tracking performance was operationalized based on the RMSE from the center point. The participants’ performance was best when they performed on their own in the stable condition (relative to the flexible condition). Team work improved tracking performance only in the flexible task condition; **B**. System Monitoring Task: system monitoring task performance was operationalized based on the mean number of misses per block; **C**. Resource Management Task: resource management task performance was operationalized based on the absolute mean deviation from optimal filling levels. **D**. Communications Task: communications task performance was operationalized based on the mean number of misses per block. In **B**, **C**, and **D** single task performance was better in the flexible than in the stable cognitive mode condition and when performed in teams, whereby the teamwork effect was larger in the stable compared to the flexible condition

The participants’ tracking performance was best explained by an interaction between *condition* and *performance mode* (*BF* = 91.40). Thus, they performed worse in the flexible condition (*M* = 0.06, *SD* = 0.02, range [0.03, 0], *M*_Bayes_ = 0.08, CI [0.07, 0.09], Tracking ~ exponentially modified Gaussian Distribution) than in the stable condition (Δ*M* = − 0.02, *SD* = 0.01, range [− 0.07, 0], Δ*M*_Bayes_ = − 0.02, CI [− 0.02, − 0.01], β ~ Gaussian Distribution). Besides, their performance improved when they performed in teams, but only in the flexible condition (Δ*M* = − 0.02, *SD* = 0.03, range [− 0.07, 0.07], Δ*M*_Bayes_ = − 0.02, CI [− 0.02, − 0.01], β ~ Gaussian Distribution), and not in the stable condition (Δ*M* = 0.02, *SD* = 0.02, range [− 0.06, 0.07], Δ*M*_Bayes_ = 0.01, CI [0.01, 0.02], β ~ Gaussian Distribution) (see Fig. 3A).

#### System monitoring task

Moreover, system monitoring task performance was best explained by an interaction between *condition* and *performance mode* (*BF* = 184.68). The participants’ solitary task performance was better in the flexible condition (*M* = 2.5, *SD* = 2.83, range [0, 19], *M*_Bayes_ = 2.58, CI [2.45, 2.69], System Monitoring ~ exponentially modified Gaussian Distribution) than in the stable condition (Δ*M* = 4.25, *SD* = 5.44, range [− 4, 21], Δ*M*_Bayes_ = 0.5, CI [0.33, 0.65], β ~ Gaussian Distribution). Besides, their performance improved when they performed in teams both in the flexible condition (Δ*M* = − 2.13, *SD* = 3.22, range [− 17, 1], Δ*M*_Bayes_ = − 0.02, CI [− 0.18, 0.13], β ~ Gaussian Distribution), and in particular in the stable condition (Δ*M* = − 4.57, SD = 5.4, range [− 21, 2], *M*_Bayes_ = − 0.5, CI [− 0.71, − 0.3], β ~ Gaussian Distribution) (see Fig. 3B).

#### Resource management task

Similarly, resource management task performance was best explained by an interaction between *condition* and *performance mode* (*BF* = 109.35), whereby the participants’ solitary resource management task performance was better in the flexible condition (*M* = 519.92, *SD* = 222.75, range [247.58, 1224.01], *M*_Bayes_ = 571.62, CI [513.40, 630.09], Resource Management ~ Gaussian Distribution) than in the stable condition (Δ*M* = 202.29, *SD* = 277.93, range [− 227.11, 1094.85], *M*_Bayes_ = 118.02, CI [53.30, 181.73], β ~ Gaussian Distribution). But their performance improved barely when they performed the task with a partner in the flexible condition (Δ*M* = − 19.98, *SD* = 215.52, range [− 463.84, 706.95], *M*_Bayes_ = − 125.78, CI [− 190.85, − 61.40], β ~ Gaussian Distribution), compared to the stable condition (Δ*M* = − 307.57, *SD* = 334, range [− 1140.82, 250.07], *M*_Bayes_ = − 136.25, CI [− 210.79, − 61.50], β ~ Gaussian Distribution) (see Fig. 3C).

#### Communications task

Also, the participants’ communications task performance was best explained by an interaction between *condition* and *performance mode* (*BF* = 6787.51). Thus, their solitary task performance was better in the flexible condition (*M* = 2.67, *SD* = 1.47, range [1, 5], *M*_Bayes_ = 2.41, CI [1.77, 3.05], Communication ~ exponentially modified Gaussian Distribution) than in the stable condition (Δ*M* = 2.45, *SD* = 2.47, range [− 1.5, 8], *M*_Bayes_ = 1.1, CI [0.43, 2.00], β ~ Gaussian Distribution). Besides, their performance improved when they performed with a partner both in the flexible condition (Δ*M* = − 2.38, *SD* = 1.31, range [− 5, 0.5], *M*_Bayes_ = − 187, CI [− 2.71, − 0.99], β ~ Gaussian Distribution), and in in the stable condition (Δ*M* = − 2.61, *SD* = 2.58, range [− 9, 1], *M*_Bayes_ = − 1.17, CI [− 2.19, − 0.42], β ~ Gaussian Distribution) (see Fig. 3D).

### Task load

#### Conditional differences in task load

Neither *condition*, *performance mode* nor an interaction between both affected how much task load the participants perceived (*BF*s < 0.86). When the participants performed the openMATB in the flexible condition and on their own (solitary), they displayed an average TLX score of 1.11 (*SD* = 0.33, range [0.09, 1.62], *M*_Bayes_ = 1.17, CI [1.07, 1.26], TLX ~ Gaussian Distribution). The TLX score was equally large in the stable condition (Δ*M* = 0, *SD* = 0.37, range [− 1.5, 0.74], Δ*M*_Bayes_ = − 0.02, CI [− 0.12, 0.07], β ~ Gaussian Distribution) but 0.08 times larger when they performed as a team (*SD* = 0.57, range [− 0.98, 2.29], Δ*M*_Bayes_ = − 0.06, CI [− 0.16, 0.04], β ~ Gaussian Distribution). Besides, when the participants performed the openMATB in the stable condition in teams, the TLX score was smaller by − 0.06 (*SD* = 0.56, range [− 2.36, 1.63], Δ*M*_Bayes_ = 0.03, CI [− 0.09, 0.14], β ~ Gaussian Distribution).

#### Multitasking performance and task load

**Fig. 4:**
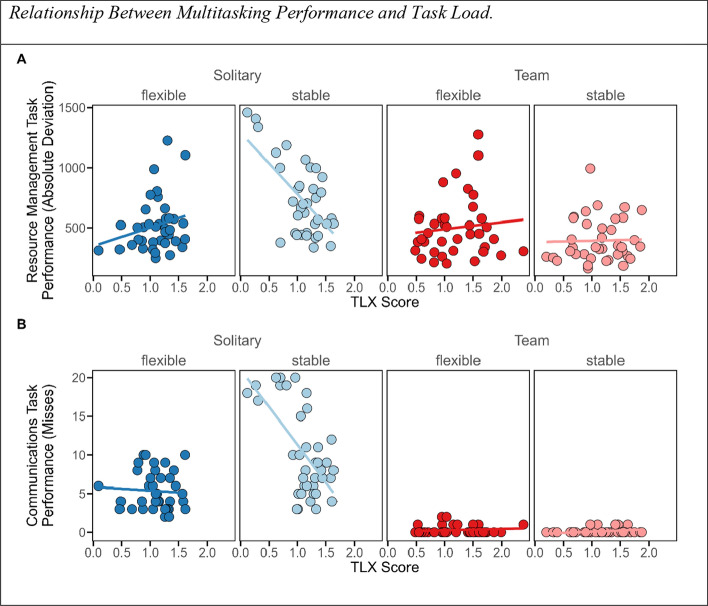
Relationship between multitasking performance and task load. *Note.* Relationship between perceived task load (TLX score) and performance in the resource management task (as a function of the absolute mean deviation from optimal filling levels) **A** and the Communications Task (based on misses), **B** in four conditions: differential cognitive mode conditions are indicated in two levels of transparency (flexible: opaque, stable: translucent), and differential performance mode conditions in two different levels of color (solitary: blue vs. team: red). The participants performed better in the tasks the more task load they experienced but only in the stable control condition and when they performed without a partner. In the remaining conditions, associations were non-existent or negligibly small

Nevertheless, experienced task load appears to play a role in predicting resource management (*BF* = 3.91) and communications task performance (*BF* = 19.36). Thus, the participants performed better in the solitary condition, the more task load they experienced but only in the stable condition. This was not the case when they performed in teams or in the flexible condition (see Fig. 4, for a visualization of the effect, and Table 1 for a summary of all regression coefficients).

**Table 1:** Impact of task load, condition and performance mode on resource management and communications task performance

	Resource management task performance				
	*M*	*SE*	*M*_Bayes_	95% *CI*	
				*LB*	*UB*
Intercept (flexible + Solitary)	347.85	102.42	587.61	490.37	685.12
Condition (stable)	944.46	125.25	185.1	106.63	262.91
Performance mode (Team)	84.99	121.79	− 150.29	− 227.39	− 72.08
Condition (stable) x	− 995.48	161.92	− 159.11	− 241.52	− 76.89
Performance mode (Team)					
TLX	155.51	85.65	− 24.39	− 98.23	50.01
TLX x condition (stable)	− 669	108.55	− 52.98	− 122.43	15.91
TLX x performance mode (Team)	− 99.03	102.94	53.96	− 14.04	121.83
TLX x condition (stable) x	623.9	137.05	− 18.27	− 95.02	58.56
Performance mode (Team)					
	Communications Task Performance				
Intercept (flexible + Solitary)	5.87	1.41	5.3	3.79	6.8
Condition (stable)	15.21	1.84	3.06	1.65	4.52
Performance mode (Team)	− 5.8	1.75	− 3.99	− 5.43	− 2.54
Condition (stable) x	− 15.52	2.37	− 2.61	− 4.14	− 1.05
Performance mode (Team)					
TLX	− 0.48	1.22	− 1	− 2.28	0.29
TLX x condition (stable)	− 9.29	1.59	0.3	− 0.95	1.54
TLX x performance mode (Team)	0.66	1.47	− 0.86	− 2.08	0.34
TLX x condition (stable) x	9.43	2	− 0.73	− 2.09	0.65
Performance mode (Team)					

*M*: estimated averages based on hierarchical linear regression analysis; *M*_Bayes_: Bayesian posterior distribution estimate; CI: Credibility interval; *LB*: lower bound; *UP*: upper bound

### Control analyses

Additional analyses concerning team performance in the stable condition relative to single and flexible task performance in the openMATB did not indicate that any demographic measure or questionnaire scale score explained a sufficiently large portion of the observed openMATB interaction effects. The best-supported model comprised resource management task performance and military service duration. However, there was only anecdotal evidence in favor of a relationship between these variables, *BF* = 2.85 (< 3).

## Discussion

We investigated if and how multitasking performance in a low-fidelity flight simulator (openMATB) changes depending on the cognitive control mode (stable vs. flexible) and the operation mode (solitary vs. team) being employed. Our results consistently indicate that single-task performance differed depending on whether participants were instructed to operate the openMATB in a *flexible* relative to a *stable* control mode (Stasch & Mack, 2023a): when instructed to prioritize the tracking task in the stable condition, tracking performance improved. Likewise, performance in the remaining (secondary) tasks declined. Thus, our data were in line with *H1a*. Furthermore, the opposite effect could be observed in the flexible condition where the cadets displayed better performance in the secondary tasks, while tracking task performance decreased (relative to the stable condition), as predicted by *H1b*. Additionally, we expected that the cadets’ tracking performance in the stable condition might improve with the aid of a partner. However, this was not the case, which opposes *H2a*. In contrast, participants performed much better in the secondary tasks in teams in the stable condition (as predicted by *H2b*) and in the flexible condition. The latter also applied to the tracking task. Thus, our data were also in line with *H2c* and *H2d*. Moreover, an exploratory analysis indicated a relationship between solitary resource management and communications task performance and perceived workload in the stable condition, respectively.

### Effects of teamwork on multitasking performance

Thus, our data clearly demonstrate that we were not only able to bias individuals towards employing either a stable or flexible strategy to either focus on a primary (tracking) task or to attend to all tasks equally (and thereby replicating previous effects on the role of the stability-flexibility dilemma in multitasking (Stasch & Mack, 2023a)), respectively; but also that our outcomes align with the predictions made by the theoretical framework outlined at the beginning of this report (Goschke, 2003, 2013). This raises the question if our data therefore also support the idea of a working memory updating threshold as underlying mechanism of these results? Given that we did not assess the individuals’ working memory abilities we can neither confirm nor reject this notion.

However, we might speculate on the implications of associations between perceived workload and openMATB performance in this regard. The updating threshold is supposed to shield internal representations from distractions – especially in the stable condition. Thus, it makes sense that better openMATB performance in the stable solitary condition goes along with more perceived workload as this could implicitly indicate more effort to suppress irrelevant and to focus more on relevant information. Furthermore, given that the resource management task and communications task illustrated the only tasks actually requiring to memorize information or a task schedule (thereby displaying a working memory requirement), the association between perceived workload and these task performances could indirectly support the idea of a working memory updating threshold predicting multitasking performance. However, we would like to point out that this is a post-hoc speculation and further empirical research will be required to confirm this hypothesis.

Aside this, we found that teamwork improved the participants’ performance across all conditions except tracking performance in the stable condition (where there were even indications of performance decrements). Moreover, it should be noted that the performance in the resource management task was comparatively small in the flexible task condition than in the other tasks. Thus, teamwork only supported task performance if actually needed, e.g., in the flexible condition where support was required to achieve peak performance (Mathieu et al., 2000; Tremblay et al., 2012). But the slight tracking task performance decrements in the stable condition also indicate that managing a partner can become a liability if all attention resources are focused towards one task (Bowers et al., 1997; Urban et al., 1996). Picking up on this, a Partner 2 may impair Partner 1’s tracking task performance in the stable condition by interfering with internal shielding e.g., by requesting consulting, offering advice, and/or socializing. Following that logic, Partner 1’s performance may be better in these conditions when left alone. With this being said, one might also wonder know why resource management task performance was only slightly improved in the flexible task condition? We speculate that this effect may be related to the task requirements of the resource management task and our experimental design: most of the individuals reported that their strategy to solve the resource management task was to set the pumps at the beginning of the trial and to focus on the remaining task until significant changes were required. Given that Partner 1 nevertheless pressed the buttons for this, and Partner 2 only monitored the flow of the pumps, there was not much gain possible in teamwork in the resource management task performance.

Picking up on that, we would like to highlight that the participants were allowed to discuss strategies on how to perform the tasks at any time. They were only required to continuously perform the tracking task together to ensure that they performed as teams throughout the task. Individual agreements on strategies and how they complied with them certainly affected their performance. We controlled for such effects using questionnaire scores. But there were no indications for confounds by personality and/or social traits. Nevertheless, describing and classifying such strategies, for instance by speed data analysis or open-ended questions, should be taken on in future research.

Thus, we conclude from our data that teamwork has the potential to significantly improve multitasking performance – but only if there is room for improvement.

### Implications for the design of adaptive assistance systems

There is only little research on the effects on teamwork on aviation performance. Thus, our empirical results contribute valuable insights into contextual factors in aviation performance and may be used to improve working conditions, for instance, by developing adaptive assistance systems accordingly. Adaptive assistance systems can dynamically allocate task responsibilities between systems and human operators based on the diagnosis of the current user state (Schwarz & Fuchs, 2017). The modification of function allocation, for instance, in the form of *task sharing* or *task offloading*, is one possibility to assign whether the machine or the human operator is responsible in performing a function, a task, or a subtask (Feigh et al., 2012). Since most cognitive adaptive assistance systems only consider the user state of one operator, we argue, based on our results, that this taxonomy should also consider the presence of an additional operator in the process of function allocation. In the following, the current user state refers to the cognitive control mode of one operator, which triggers adaptations that are described in the following.

#### Recommendations on function allocation of continuous, manual tasks

Our results indicate that teamwork performance only improved in the flexible condition for the tracking task. Therefore, we argue that if the main operator (i.e., pilot flying) is in a stable control state, the additional operator should not have any task responsibility for a continuous manual tracking task, similar to a *task sharing* function allocation. If the main operator is in a flexible control mode, the additional operator should also have some kind of responsibility for the tracking task, since our results indicate that this instruction increased tracking task performance in comparison to the single-flexible condition.

#### Recommendations on function allocation of stimulus–response tasks

Regarding stimulus–response tasks requiring quick manual reactions, similar to the system monitoring task or the communication task, we recommend assigning responsibility for these tasks to both operators. Our results demonstrated that teamwork improved performance in those tasks compared to the single condition, especially in the stable condition. Assuming that one subject focused on the tracking task in the stable condition, the other subject might have compensated for this imbalance of attention allocation. Consequently, we recommend to assign task responsibility in a *task offloading* manner to the additional operator when the main operator is in a stable control mode.

#### Recommendations on function allocation of strategic decision-making tasks

The resource management task is representative of a strategic decision-making task. For that task, performance was better in the flexible condition compared to the stable condition when performed in the single condition. Adding a partner only improved performance in the stable condition, but barely in the flexible condition. Therefore, we recommend assigning task responsibility to an additional operator in a *task offloading* manner when the primary operator is in a stable control state. This should avoid interferences between both operators, who may both have focused on the task in the flexible condition.

#### Recommendations on function allocation in multitasking tasks

To this point, our recommendations related to specific task requirements. Assistance systems fine-tuned to such requirements however may only provide limited support. Accounting for more general characteristics, e.g., user capacity, may constitute a promising solution to this. Fox et al. (2021) introduced a metric, the *multitasking throughput* (MT), which can be used for such purposes, given that it illustrates a standardized measure of alterations of cognitive capacity accounting for task performance of each task in a multi- compared to a single-task condition based on a reverse hazard function model. Recently, it has also been successfully used to operationalize differences in multitasking performance between individuals and teams (Fox et al., 2025). Unfortunately, this approach was not applicable for our research purpose as we were explicitly interested in how teamwork would affect the stability-flexibility dilemma. Hereby, conditional subtask as opposed to general task performance differences needed to be considered. In theory the MT could somehow accommodate for that by means of task performance weighting. But we had too little prior knowledge on the anticipated effects for applying the MT in this regard. Furthermore, the MT is based on one type of performance measure (Fox et al., 2021). However, we used three different performance measures to investigate multitasking Future research could address this issue by operationalizing task performance of each task based on reaction time data only. However, this was not applicable in our case, given that the individuals predominantly missed events in the solitary and stable task instruction condition in the system monitoring and communications tasks (and thus there were no responses towards the events). Future research would need to fundamentally alter the MATB scenarios to motivate a different behavior.

### Limitations on generalisability

With this being said, we would also like to point out that our effects unlikely fully translate to real-life piloting conditions. Participants of this study performed a highly standardized low-fidelity flight simulation with “only” four tasks in a university laboratory obviously preventing individuals from experiencing authentic piloting conditions. Furthermore, the experimental design with two individuals sitting side-by-side and sharing the tracking task does significantly differ from conditions experienced by a pilot flying and a pilot monitoring. In addition to that, only some individuals had acquired flight experience prior to our investigation, while pilots flying aircrafts completed an entire training. Thus, our effects of teamwork probably apply foremost to conditions as experienced by pilots in training. Future research should therefore also focus on interaction effects between experience/expertise and teamwork in flight performance to gain further insights into this.

Furthermore, some methodological aspects may have confounded the participants behavior. The feedback score, for instance, was provided only during the individual performance assessment, while no performance feedback was given during the team assessment. Furthermore, the gamification approach, which framed poor weather conditions as a justification for promoting the stable control mode, did not alter the actual difficulty of the tracking task. Task difficulty was deliberately kept constant across the flexible and stable conditions to eliminate it as a confounding factor. However, in real-world scenarios, adverse weather conditions inherently increase the difficulty of tracking and other tasks, which may lead to different cognitive control strategies than those observed in the present study.

## Conclusion

To conclude, the current study underscores the importance of considering the cognitive control mode of an operator and teamwork when performing multitasking in a flight environment. Counterintuitively, teamwork may not always be beneficial – rather this effect appears to be highly task-specific and dependent on the cognitive control mode of the main operator (that is when support is actually needed).

## Data Availability

The datasets generated and analyzed during the current study, code, and supplementary materials are available in the following OSF repository (https://osf.io/mbp43/).

## Abbreviations

BF: Bayes factor
CFI: Comparative fit index
CI: Credibility interval
openMATB: Open-source version of the multi-attribute task battery
RMSE: Root mean squared error
TLX: NASA task load index
